# Integrating Radiology and Point-of-Care Ultrasound Training in Internal Medicine Residency: A Modern Imperative for Patient-Centered Care

**DOI:** 10.7759/cureus.108482

**Published:** 2026-05-08

**Authors:** Abdelrazig Suliman

**Affiliations:** 1 Internal Medicine, University of Texas Health Science Center at Tyler, Tyler, USA

**Keywords:** clinical decision-making, diagnostic imaging, internal medicine residency, medical education, patient-centered care, point-of-care ultrasound (pocus), radiology education

## Abstract

The rising complexity of patient care, together with growing imaging dependency, requires internal medicine residents to develop essential radiologic competencies. The majority of internal medicine (IM) programs lack sufficient structured education about imaging interpretation and point-of-care ultrasound (POCUS) training. The paper advocates for IM residency training to include a formal radiology and POCUS curriculum because it enhances diagnostic accuracy, clinical decision-making, and patient-centered outcomes. The contemporary clinical practice has seen physical examination skills fade away while healthcare providers increasingly rely on diagnostic imaging. The limited formal training IM residents receive for interpreting imaging studies leads to both underutilization and misinterpretation of these studies. The diagnostic tool POCUS provides an efficient, accessible, and cost-effective method to enhance clinical evaluation. The implementation of structured education programs that combine didactics with bedside application and faculty mentorship will connect traditional clinical competencies to modern diagnostic requirements. The integration of radiology and POCUS training into IM residency programs serves as an essential addition to patient care instead of replacing the physical examination. A structured curriculum enables residents to make safer and more informed decisions, especially when they work in high-acuity and resource-limited settings.

## Editorial

Introduction

Internal medicine residency training stands at a critical decision point. Medical practitioners must now process an unprecedented amount of data as medical knowledge grows rapidly, patient complexity increases, and technological advancements emerge. Residents' physical examination skills show a declining trend, while traditional clinical abilities receive increasing attention from the medical community. The central role of imaging in clinical decision-making has not been matched by equivalent growth in formal education about radiologic interpretation and point-of-care ultrasound (POCUS) skills [1,2]. This article presents an evidence-informed perspective on the integration of radiology and POCUS training into internal medicine residency. Drawing on existing literature and educational frameworks, it aims to highlight current gaps and propose a structured, competency-based curriculum to address evolving clinical demands.

The problem

Teaching bedside medicine and physical skills has declined over the past few decades. In the 1960s, almost 75% of internal medicine residency training happened at the bedside, but now only 16% to 20% of training takes place there [3,4]. Several studies have raised concerns about the physical diagnosis skills of internal medicine house staff. One study found that they correctly identified less than 20% to 40% of important cardiac findings [5]. A recent review of inpatient charts over four decades showed that junior house staff now document fewer and less detailed physical exam findings and rely more on lab tests and imaging [6]. These results suggest that less focus on bedside teaching and more reliance on technology have led to lower confidence in physical diagnosis among internal medicine residents.

Although imaging plays a central role in contemporary clinical decision-making, formal radiology education within internal medicine residency programs remains limited and inconsistently structured. Current training is frequently fragmented, delivered through isolated lectures without longitudinal integration or competency-based assessment [1,2]. Consequently, many residents lack confidence in interpreting common imaging studies, which may lead to misinterpretation, delayed diagnoses, and unnecessary additional testing. Training opportunities vary substantially across institutions, and the lack of standardized instruction hinders consistent adoption [7,8]. This deficiency is particularly concerning given evidence that POCUS improves diagnostic accuracy, reduces time to diagnosis, and enhances clinical decision-making [8].

These findings show that traditional internal medicine training is not keeping up with the growing use of imaging in today’s clinical practice. Even though radiology and point-of-care ultrasound (POCUS) are used more often in patient care, many residents still do not get enough structured training to interpret imaging studies or use ultrasound at the bedside. Because of this, trainees may feel less confident using these tools in real clinical situations, which can lead to delays in diagnosis, slower decision-making, and differences in patient outcomes.

The role of radiology in modern internal medicine

The management of various internal medicine conditions heavily depends on radiologic imaging techniques. Internists need to interpret imaging results regularly to make proper decisions when assessing patients who experience chest pain, shortness of breath, abdominal pain, infection, etc. The ability to read basic chest radiographs, interpret CT and MRI cross-sectional imaging, and identify important pathological patterns leads to better diagnostic accuracy and reduced specialist consultation needs [1]. Physicians who understand imaging study indications and limitations, as well as risks, can practice more judiciously by minimizing unnecessary radiation exposure and reducing healthcare costs [2]. 

The COVID-19 pandemic brought significant changes to medical practice, as healthcare providers faced both diagnostic challenges and concerns about virus transmission at the outset. Medical staff reduced or eliminated traditional clinical assessment methods, including auscultation and physical examinations, to minimize healthcare worker exposure and reduce patient contact duration [7,9]. The situation required diagnostic radiology to become an essential diagnostic tool for evaluation and management purposes [8].

The medical diagnosis and patient monitoring of COVID-19 involved using chest radiography (CXR) and computed tomography (CT) scans as primary diagnostic tools. CT imaging showed excellent results in detecting typical lung findings through ground-glass opacities and consolidations before RT-PCR confirmation and without clear clinical symptoms [10-12]. Radiologic imaging provided essential information to doctors for diagnosing patients and for determining hospital admission needs, oxygen therapy requirements, and disease progression [11-13].

The pandemic revealed an essential training gap, as internal medicine residents needed stronger skills to interpret medical images independently. This highlighted that residents must learn essential radiological diagnostic skills, which should serve as their primary tool for treating urgent and infectious diseases. The development of these competencies will enable future physicians to provide safe and effective care during public health emergencies when standard assessment methods become restricted or hazardous [12].

The development of core imaging knowledge for internists enables efficient patient-centered care and promotes their ability to reason independently in clinical situations. Several professional organizations, including the American Institute of Ultrasound in Medicine and the Society of Hospital Medicine, emphasize the need for structured imaging and POCUS training with competency-based assessment in clinical practice [2,8].

POCUS: the “new stethoscope”

Internal medicine has adopted point-of-care ultrasound (POCUS) as a revolutionary diagnostic instrument. Although its use is expanding, studies suggest that only a minority of internal medicine residency programs have a formal structured curriculum, indicating that comprehensive and standardized training remains limited [14]. The medical applications of POCUS include cardiovascular assessment for volume status and pericardial effusion detection, pulmonary evaluation for pneumothorax and pleural effusion diagnosis, and abdominal scanning for ascites and hydronephrosis detection. The portable nature of POCUS, along with its radiation-free operation and immediate bedside data delivery, makes it an essential tool for acute and resource-constrained environments [8]. The implementation of POCUS training improves clinical accuracy while boosting patient satisfaction and trust, as patients observe their healthcare providers conducting detailed, technology-assisted evaluations. The incorporation of POCUS in residency programs leads to faster diagnosis, improved clinical decision-making, and higher resident participation, according to institutions that have adopted this technology [8]. However, its effectiveness depends on appropriate training, quality assurance, and integration with clinical judgment to avoid operator-dependent variability and over-reliance.

Proposed curriculum framework

The structured radiology and POCUS curriculum should be distributed throughout the three years of internal medicine training as follows: PGY-1: The curriculum includes basic radiographic interpretation of chest and abdominal films, as well as POCUS orientation and initial hands-on training. PGY-2: The curriculum includes imaging integration during clinical rounds and intermediate POCUS applications for cardiac, lung, and vascular scans and case-based imaging reviews. PGY-3: The curriculum includes advanced radiology and POCUS electives, formal competency assessments, and opportunities for teaching junior residents or medical students (Table 1). Curriculum delivery can include didactic lectures, simulation labs, bedside teaching, asynchronous online modules, and faculty mentorship [2].

**Table 1:** Proposed Radiology and POCUS Curriculum for Internal Medicine Residents

Training Year	Radiology Focus	POCUS Focus	Learning Objectives	Teaching Modality	Assessment Methods
PGY-1	Chest & abdominal X-ray basics	Intro to ultrasound, probe handling, image identification	- Identify normal vs. abnormal chest/abdominal radiographs - Understand basic imaging indications - Demonstrate basic probe handling and image acquisition	Workshops, online modules	Image interpretation quizzes Direct observation POCUS logbook (minimum scans)
PGY-2	CT/MRI fundamentals, clinical integration	Cardiac, pulmonary, vascular scanning	- Interpret common CT/MRI findings in clinical context - Perform focused cardiac and lung POCUS exams - Integrate imaging into clinical decision-making	Bedside teaching, case-based reviews	OSCEs Case-based assessments Faculty evaluation of scans
PGY-3	Advanced imaging, diagnostic correlation	Procedural guidance, POCUS mentorship	- Correlate imaging findings with clinical diagnoses - Use POCUS for procedural guidance - Teach junior learners	Electives, peer teaching, advanced workshops	OSCEs Teaching evaluations Portfolio/logbook review Competency sign-off

Benefits of integration

The implementation of radiology and POCUS training provides various advantages, including better diagnostic precision and improved clinical choices; shorter time needed to identify conditions and start treatments; residents gaining increased self-assurance and independence in their practice; better patient communication and satisfaction; the training prepares healthcare providers to handle both pandemic and disaster situations; and a foundation for lifelong learning and cross-disciplinary collaboration. The integration of these competencies into residency training enables programs to develop internists who can handle intricate clinical situations autonomously. Research has validated these findings through previous investigations about POCUS and radiology education in residency training [2,8].

Barriers and solutions

There are several challenges to implementing this training, especially when it comes to time. Internal medicine residency programs are already packed into a three-year period, during which residents must become competent in many specialties while managing inpatient care, outpatient clinics, procedures, board exams, and duty-hour limits. Because the curriculum is so full, adding ongoing radiology and point-of-care ultrasound (POCUS) education can be difficult. To address this, training should be offered in brief, focused sessions that fit into current academic half-days or rounds.

Another challenge is that there may not be enough faculty with imaging experience to provide consistent teaching [2]. Many residency programs were created before bedside ultrasound became common in internal medicine, so faculty training, supervision, and resident experience with imaging can vary widely. There are also practical issues, such as getting enough ultrasound machines and making sure they work with electronic medical records. Studies have also found that not having a standard curriculum, limited hands-on practice, and uneven ways of checking skills are major barriers to putting these programs in place.

One way to address these challenges is to use a modular, high-yield teaching model during existing teaching times. More instructors can be trained through faculty development and train-the-trainer programs [8]. Sharing portable ultrasound devices and having strong support from the institution for diagnostic imaging will also make the program more practical.

Conclusion

Radiology and point-of-care ultrasound (POCUS) training should be incorporated into internal medicine residency programs, as these modalities are now essential components of modern clinical practice. Mastery of these skills allows physicians to provide safer, more effective, and patient-centered care. Although the physical examination remains fundamental, the integration of imaging with clinical reasoning improves diagnostic accuracy and facilitates more informed clinical decisions.

Further research is needed to assess the effects of structured radiology and POCUS training on diagnostic accuracy, clinical outcomes, and healthcare utilization. Implementation strategies should be adapted to institutional resources, with phased integration, faculty development, and provision of imaging tools as foundational steps. Internal medicine residency programs are encouraged to adopt structured, competency-based curricula to equip residents for the evolving requirements of contemporary clinical practice.

## References

[1] Collins J (2000). Curriculum in radiology for residents: what, why, how, when, and where. Acad Radiol.

[2] Schiller PT, Phillips AW, Straus CM (2018). Radiology education in medical school and residency: the views and needs of program directors. Acad Radiol.

[3] LaCombe MA (1997). On bedside teaching. Ann Intern Med.

[4] Crumlish CM, Yialamas MA, McMahon GT (2009). Quantification of bedside teaching by an academic hospitalist group. J Hosp Med.

[5] Johnson JE, Carpenter JL (1986). Medical house staff performance in physical examination. Arch Intern Med.

[6] Oliver CM, Hunter SA, Ikeda T, Galletly DC (2013). Junior doctor skill in the art of physical examination: a retrospective study of the medical admission note over four decades. BMJ Open.

[7] Moore CL, Copel JA (2011). Point-of-care ultrasonography. N Engl J Med.

[8] Bhagra A, Tierney DM, Sekiguchi H, Soni NJ (2016). Point-of-care ultrasonography for primary care physicians and general internists. Mayo Clin Proc.

[9] Erdevir M, Kim S, Lee H (2021). COVID- 19: The final nail in the coffin for physical examination?. J Educ Eval Health Prof.

[10] Rubin GD, Ryerson CJ, Haramati LB (2020). The role of chest imaging in patient management during the COVID-19 pandemic: a multinational consensus statement from the Fleischner society. Radiology.

[11] Varadarajan V, Shabani M, Ambale Venkatesh B, Lima JA (2021). Role of imaging in diagnosis and management of COVID-19: a multiorgan multimodality imaging review. Front Med (Lausanne).

[12] Gandhi D, Jain N, Khanna K (2020). Current role of imaging in COVID-19 infection with recent recommendations of point-of-care ultrasound in the contagion: a narrative review. Ann Transl Med.

[13] Simpson S, Kay FU, Abbara S (2020). Radiological Society of North America Expert Consensus Statement on reporting chest CT findings related to COVID-19. Endorsed by the Society of Thoracic Radiology, the American College of Radiology, and RSNA - secondary publication. J Thorac Imaging.

[14] Kelm DJ, Ratelle JT, Azeem N (2015). Longitudinal ultrasound curriculum improves long-term retention among internal medicine residents. J Grad Med Educ.

